# Phytolith occluded organic carbon in *Fagopyrum* (Polygonaceae) plants: Insights on the carbon sink potential of cultivated buckwheat planting

**DOI:** 10.3389/fpls.2022.1014980

**Published:** 2022-11-11

**Authors:** Linjiao Wang, Maoyin Sheng

**Affiliations:** ^1^ Institute of Karst Research, Guizhou Normal University, Guiyang, China; ^2^ Guizhou Engineering Laboratory for Karst Rocky Desertification Control and Derivative Industry, Guiyang, China; ^3^ National Engineering Research Center for Karst Rocky Desertification Control, Guiyang, China

**Keywords:** buckwheat, phytolith, soil organic carbon, silicon, carbon sink

## Abstract

Crop cultivation has great potential to result in a phytolith carbon sink and can play important roles in the long-term stable carbon sequestration of terrestrial ecosystems. Buckwheat, an important multigrain crop with a very long cultivation history, is widely planted around the world. The phytolith carbon sink potential of buckwheat planting is still limited in the in-depth understanding of biogeochemical carbon sequestration in croplands. In order to estimate the phytolith carbon sink potential of buckwheat planting, in the present study, six species including 17 populations of *Fagopyrum* plants were selected as study materials. Firstly, their phytoliths were extracted using the wet oxidation method; then, the phytolith-occluded organic carbon (PhytOC) contents were determined using the spectrophotometry method; finally, the phytolith carbon sink potential of buckwheat planting was estimated. Results showed the following: 1) The PhytOC content range of the six *Fagopyrum* species studied was 0.006%~0.038%, which was significantly lower than that of rice, wheat, sugarcane, and some cereal and oil crops. There were significant differences in total silicon, phytolith, and PhytOC content of *Fagopyrum* plants among the different species, different organs (root, stem, and leaf), and different living forms (annual, partly perennial, and completely perennial). There were significant positive relationships between PhytOC and phytolith content and between phytolith and total silicon content. 2) The average phytolith carbon sequestration rate of *Fagopyrum esculentum* and *Fagopyrum tataricum* planting was 2.62 × 10^-3^ and 1.17 × 10^-3^ t CO_2_ hm^-2^·a^-1^, respectively, being approximately equal to that of terrestrial shrub vegetation. 3) The global total amount of phytolith carbon sequestration of buckwheat planting reached 5,102.09 t CO_2_ in 2018, and the Chinese total amount of phytolith carbon sequestration of buckwheat cultivation was 624.79 t CO_2_ in 2020. The phytolith carbon sink of buckwheat planting had significant potential for playing obvious roles in the carbon cycle. The present results are of great significance in crop phytolith studies and provide important references for phytolith carbon sink potential estimation of farmland ecosystems.

## Introduction

Phytoliths are the amorphous silica minerals (SiO_2_·nH_2_O) originating from the soluble monosilicic acids (H_4_SiO_4_) that are absorbed from soils by plant roots and gradually formed in cell walls or intercellular spaces of plant stems, roots, and leaves in the process of plant transpiration (Parr and Sullivan, 2005; Hodson, 2016). In the formation process of phytoliths in plants, a certain amount of organic carbon was occluded in phytoliths, which is called phytolith-occluded organic carbon (PhytOC) (Song et al., 2012; Parr and Sullivan, 2014). Phytoliths exhibit strong resistance against corrosion decomposition and oxidation. Even if plants have been dead, decayed, and combusted, phytoliths still can be stored in soils or sediments for a very long time (Parr and Sullivan, 2005), likely being preserved in soil microaggregates (Li et al., 2020; Li et al., 2022). As protected by external phytoliths, PhytOC is more stable than most organic carbon fractions. Especially, a part of PhytOC can be reserved in soils for thousands to 10,000 years (Parr and Sullivan, 2011). It is reported that soil PhytOC can account for 82% of the total organic carbon in some soils after more than 2,000 years of soil organic carbon decomposition (Parr and Sullivan, 2005; Gherardi and Sala, 2020). Phytolith carbon sink, as a kind of long-term stable biogeochemical carbon sink mechanism, has raised the attention of global scientists. Although there still are some controversies in phytolith carbon sink potentials (Hodson, 2019), this kind of carbon sequestration mechanism in thousand years’ timescale by plants has become a hot spot and an extremely important content for long-term stable carbon fixation research in terrestrial ecosystems (Song et al., 2014; Chen et al., 2018; Huang et al., 2020).

Buckwheat is an herbaceous plant of the genus *Fagopyrum* in the Polygonaceae family. There are 23 species reported in this genus, two of which are cultivated for food crops: *Fagopyrum esculentum* and *Fagopyrum tataricum* (Sheng et al., 2011; Sheng et al., 2013). Buckwheat is an important multigrain crop with remarkable nutritional and healthcare values. Buckwheat seeds are not only comprehensive in nutrition but also rich in highly active medicinal ingredients (Chen et al., 2004). Buckwheat has a very long cultivation history and is planted widely in the world. After thousands of years of transmission and planting, cultivated buckwheat has spread to Asia, Africa, North America, South America, Europe, and Oceania (Sheng et al., 2011). According to statistics from the United Nations Food and Agriculture Organization (http://fao.org./home/zh), the global planting area of cultivated buckwheat has reached 3.2 × 10^6^ hm^2^ in 2018 with a total seed output of 3.83 × 10^6^ tons. In 2020, the Chinese-cultivated buckwheat planting area was 3.70 × 10^5^ hm^2^, being mainly distributed in Inner Mongolia, Shaanxi, Gansu, Ningxia, Shanxi, Yunnan, Sichuan, and Guizhou (Chinese National Bureau, https://data.stats.gov.cn/). In addition, in recent years, since buckwheat can grow well in poor soils, it has been widely used for the vegetation restoration of degraded ecosystems (Sheng et al., 2011).

For a long time, due to the lack of understanding of phytolith carbon sequestration, it has been believed that the carbon sink of herbal crops is basically zero because organic substances of roots, stems, and leaves will quickly rot and decompose after harvesting (Li et al., 2013; Guo et al., 2015; Nguyen et al., 2019). With further studies on phytoliths in recent years (Hodson et al., 2005; Fishkis et al., 2009; Guo et al., 2015; Li et al., 2019), it has become a consensus that there are huge potentials in having a phytolith carbon sink in herbal crops. The phytolith carbon sequestration flux of rice planting is 67.80 kg CO_2_ hm^-2^·a^-1^, significantly higher than that of some terrestrial natural ecosystems of wetlands, forests, and shrubs (Song et al., 2016; Qi et al., 2021). In addition, the carbon fixation stock of Chinese rice phytoliths is 2.04106 t CO_2_ per year, also higher than that of the terrestrial natural ecosystems of forests and shrubs (Zuo and Lü, 2011; Schaller et al., 2019). It has been widely recognized that phytolith carbon sequestration of herbal crops is an important component of long-term stable terrestrial carbon sinks and has great contributions to terrestrial vegetation carbon sinks (Zuo et al., 2014; Yang et al., 2016; Yang et al., 2018; Li et al., 2020; Song et al., 2022). In recent years, the significant carbon sink potential of wheat, maize, millet, and sugarcane has been reported successively, proving that grass family crops have high silicon (Si) content and carbon sink potential (Zuo and Lü, 2011; Song et al., 2013). However, to date, phytolith studies on non-grass family crops are scarce.

As a globally important grain crop with a very long planting history and extensive planting area, there is still no report on buckwheat phytolith and PhytOC studies. Consequently, the carbon sink potential of buckwheat phytoliths is still in the dark, seriously limiting the in-depth understanding of crop phytolith carbon sequestration and hindering the accurate estimation of whole farmland ecosystem phytolith carbon sink potentials. It can be assumed that *Fagopyrum* plants contain a certain amount of phytoliths and PhytOC, and cultivated buckwheat planting has obvious phytolith carbon sink potential and can play a significant role in carbon cycle regulation. To verify this hypothesis, in the present study, phytoliths and PhytOC of six species including 17 populations of *Fagopyrum* plants were extracted and determined, respectively, and the phytolith carbon sink potential was estimated. The present results can provide references for the accurate estimation of farmland ecosystem phytolith carbon sink potential.

## Materials and methods

### Plant material

Seventeen populations of buckwheat plants involving six *Fagopyrum* species, that is, *F. esculentum*, *F. tataricum*, *F. tatari-cymosum*, *F*. *cymosum*, *F. megaspartanium*, and *F. pilu*s, were selected as the present plant materials. All plant materials were planted in Anshun fields of Guizhou Province, China. The details of plant materials were listed in **Table 1**.

**Table 1:** Plant materials studied.

Species	Populations	Native to	Living form
*F. esculentum* Moench	Hongtian 5#	Guizhou, China	Annual
	Hongtian 2#	Guizhou, China	Annual
	Tianqiao BH	Jilin, China	Annual
*F. tataricum* (L.) Gaertn	Heimi-15	Guizhou, China	Annual
	Kuqiao YZX	Sichuan, China	Annual
	Yunku 1#	Yunnan, China	Annual
	GMK 2010-2002#	Guizhou, China	Annual
	GMK 2012-2163#	Guizhou, China	Annual
	GMK 2012-19#	Guizhou, China	Annual
*F. tatari-cymosum* QF Chen	GJK 2012-2294#	Guizhou, China	Partly perennial
	GJK 2012-2298#	Guizhou, China	Partly perennial
	GJK 2012-1206#	Guizhou, China	Partly perennial
*F. megaspartanium* QF Chen	JQ 1#	Xizang, China	Completely perennial
	JQ 3#	Guizhou, China	Completely perennial
	Low JQ	Sichuan, China	Completely perennial
*F. pilu*s QF Chen	MYQ 1#	Xizang, China	Completely perennial
*F*. *cymosum* (Trev. Meisn.) QF Chen	HXJQ	Sichuan, China	Completely perennial

### Plant and soil sampling

Buckwheat samples of three different organs (roots, stems, and leaves) were collected during the two seasons, in May (flowering period) and July (seed matured period). Plant samples collected were rinsed twice with tap water, ultrasonic water cleaned (100 Hz) for 5 min, dried at 70°C to constant weight, crushed, and screened using 0.25-mm sieves, successively, for phytolith extraction. Planting plot soils and rhizosphere soils were sampled at the same time. Plot soils were sampled in three different soil profiles, 0–5 cm, 5–10 cm, and 10–20 cm, respectively. Rhizosphere soils (soils within a radius of 4 mm around the roots) were collected by the soil shaking method (Xiao et al., 2022). All soil samples were naturally dried in dark dislodged plant and animal residues and crushed for the determination of soil physical and chemical properties. A minimum of three replicates were collected for each type of samples and plants.

### Measurement of soil physical and chemical properties and plant total silicon content

Soil bulk density (BD), natural water content (NWC), field moisture capacity (FMC), pH, total soil organic carbon (TSOC), total nitrogen (TN), total phosphorus (TP), and available Si content were determined. BD, NWC, and FMC were measured by the ring knife method (Sheng et al., 2018). Soil pH was determined in 1:2.5 w/v mixtures of soil and distilled water by a pH meter. TSOC, TN, and TP contents were measured using the potassium dichromate oxidation–ferrous sulfate titrimetry method, Kjeldahl method, and molybdenum antimony colorimetric method, respectively (Sheng et al., 2018). Soil-available Si content was determined using the citric acid buffer (0.025 mol·L^-1^) extraction–molybdenum blue colorimetric method (Li et al., 2021). Plant total Si content was determined using the lithium metaborate melting–nitric acid buffer extraction–molybdenum blue colorimetric method (Li et al., 2014).

### Phytolith extraction

Phytoliths were extracted using the wet oxidation method modified from Yang et al. (2018). The procedure included mainly the following: 1) about 10-g samples were deflocculated with 5% sodium polyphosphates, and the supernatant was collected; 2) H_2_O_2_ (30%) and HCl (10%) were used to oxidize organic matter; 3) fractions (<250 μm) were separated by wet sieving and disaggregated from the organic matter by ultrasonic treatment; 4) after being oxidized secondly by HNO_3_ and KClO_3_ and centrifuged, phytoliths were extracted by heavy liquid (ZnBr_2_) with a density of 2.3 g·cm^-3^; 5) extracted phytoliths were further sieved at 7 μm, reacted by HClO_4_ for 20 min, and dried.

### Phytolith morphology observation

After being washed by distilled water centrifugation three times and washed by anhydrous alcohol centrifugation one time, the extracted phytoliths were made into slides and sealed by neutral resin. Microscopic identification was performed using an Olympus BX51 microscope (Japan) and photographed using an Olympus DP72 microphotographic system (Japan). More than 100 phytoliths of each plant material were observed and were counted by shape types. The morphologic classification of phytoliths was performed according to the standard of Ma (2003) and International Phytolith Code Nomenclature 2.0 (IPCN 2.0) (Neumann et al., 2019).

### Phytolith-occluded organic carbon content determination and calculation

PhytOC content was determined using the spectrophotometry method (Yang et al., 2014). Briefly, about 0.01-g dry phytoliths extracted were weighed and dissolved for 12 h in 0.5 ml NaOH solution (10 mol·L^-1^). Then, 1.0 ml 1/6 K_2_Cr_2_O_7_ standard solution (0.80 mol·L^-1^) and 4.6 ml concentrated H_2_SO_4_ were added and shaken up, successively. After being bathed for 1 h in 98°C water, cooled in air, and centrifuged at 3,000 rpm for 10 min, PhytOC mass was determined using spectrophotometry (Shimadzu, UV 1900, Japan) at 590 nm. Phytolith and PhytOC contents were calculated by the two following equations (Li et al., 2021): 1) Phytolith content (g·kg^-1^) = phytolith mass (g)/plant sample mass (kg); and 2) PhytOC content (g·kg^-1^) = PhytOC mass (g)/plant sample mass (kg).

### Potential estimation and geographical distribution of phytolith carbon sink

Mature individuals of *F. esculentum* and *F. tataricum* plants were randomly collected and dried. The dry biomass of roots, stems, and leaves was weighed and obtained. The phytolith carbon sequestration rate (*R*_p_) and the annual total amount of phytolith carbon sequestration (*A*_p_) were calculated by the two following equations:


(1) $$R_{p}=M\times N\times H\times \;(M_{CO}2/\;M_{C})$$

(2) $$A_{p}=R_{p}\times S$$

In the equation, *R*_p_ is the phytolith carbon sequestration rate (kg CO_2_ hm^-2^·a^-1^), *A*_p_ is the annual total amount of phytolith carbon sequestration (kg CO_2_ a^-1^), *M* is the biomass per individual plant (kg), *N* is the planting density (individual plant number per hectare), *H* is the PhytOC content (g·kg^-1^), *S* is the planting area (hm^2^), and M_CO2_ and M_C_ are CO_2_ and C molecular weight, respectively. According to field investigations and governmental statistical data, a planting density of 1,200 thousand individuals per hectare was used. The Chinese and global buckwheat planting areas referred to the statistics of the Chinese National Bureau (https://data.stats.gov.cn/) and United Nations Food and Agriculture Organization (http://fao.org./home/zh), respectively. The most recent statistical data on the buckwheat planting area of China and the world in these two institutions are 2020 and 2018, respectively. The *A*_p_ geographical distribution was made by the software ArgGIS 10.1.

### Statistical analysis

Statistical analyses were conducted by Microsoft Excel 2013 and SPSS 23.0. The significant difference between variables was tested by one-way analysis of variance (ANOVA) and the Duncan test. Before the variance analysis was conducted, the normality and homogeneity of variances were tested. Datasets that did not meet the normality and homogeneity were transformed by the natural logarithm method. Pearson method and partial correlation analysis were used to analyze the correlation between variables.

## Results

### Phytolith morphology in *Fagopyrum* plants

**Figure 1:**
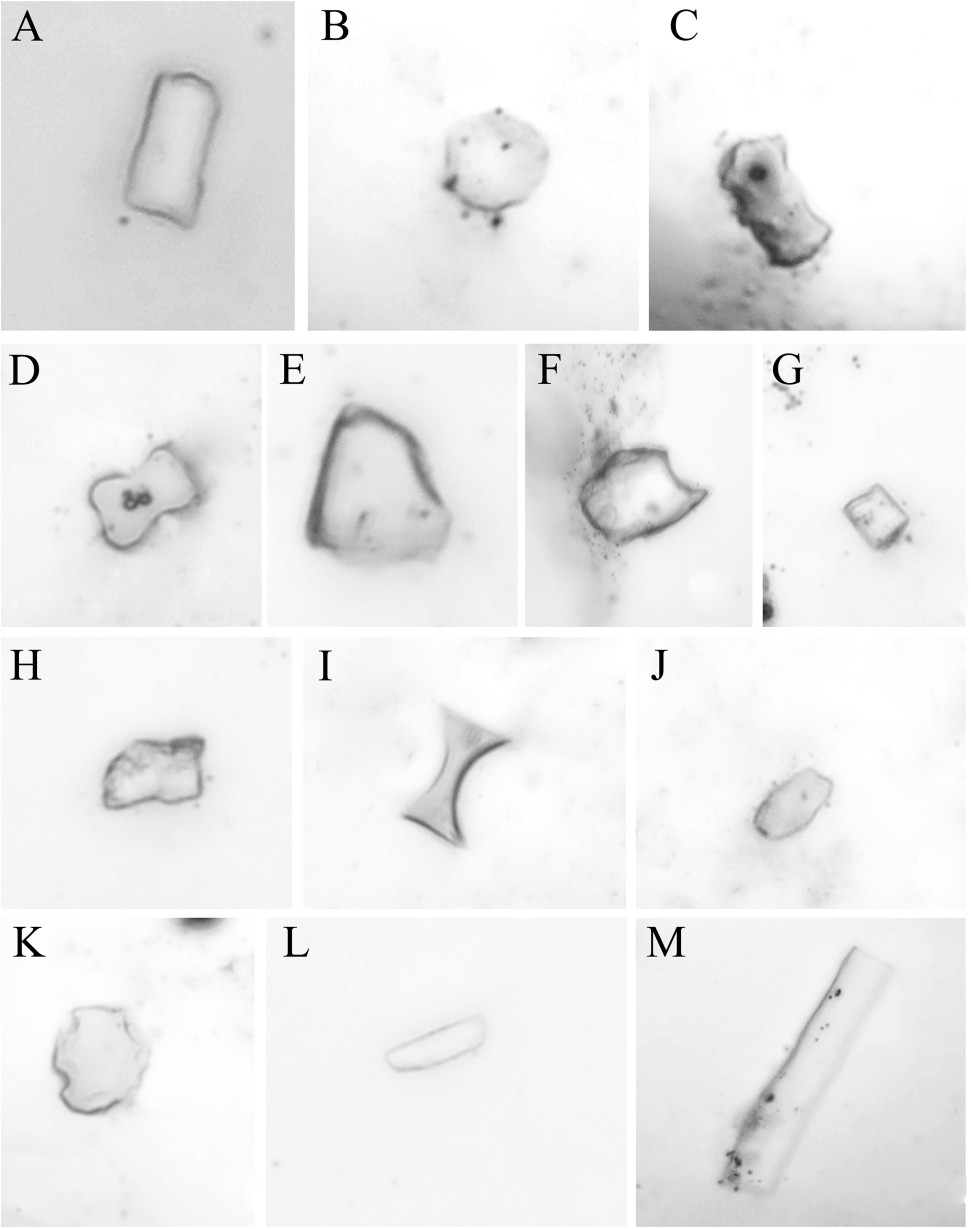
*Fagopyrum* plant phytolith shapes of **(A)** rectangle, **(B)** rotundity, **(C)** bar shape with humps, **(D)** dumbbell shape, **(E)** fan shape, **(F)** ball sharp with tips, **(G)** square, **(H)** polygon, **(I)** saddle sharp, **(J)** oval, **(K)** spin ball shape, **(L)** short bar shape, and **(M)** bar shape.

Phytolith shapes of *Fagopyrum* plants were varied, and 13 types of phytoliths were observed in the 17 populations of *Fagopyrum* plants studied, including eight main types of rotundity, short bar shape, polygon, ball sharp with tips, square, rectangle, oval, and bar shape, and five infrequent types of bar shape with humps, dumbbell shape, fan shape, saddle sharp, and spin ball shape (**Figure 1**). There was no obvious difference in shape types of phytoliths between the different species studied, and all of the above 13 shape types of phytoliths were observed in each species. In addition, among the different organs of root, stem, and leaf, among the different living forms of annual, partly perennial, and completely perennial, or among different growth periods of flowering and seed matured, no obvious difference in shape types of phytoliths was present.

### Total silicon, phytolith, and phytolith-occluded organic carbon contents in *Fagopyrum* plants

#### Total silicon, phytolith, and phytolith-occluded organic carbon contents between species

**Figure 2:**
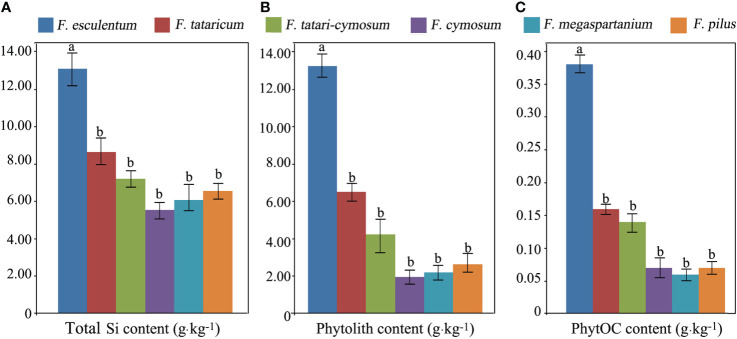
Total silicon (Si) **(A)**, phytolith **(B)**, and phytolith-occluded organic carbon (PhytOC) **(C)** content between species of *Fagopyrum* plants.

The average total Si content of the six *Fagopyrum* species of *F. esculentum*, *F. tataricum*, *F. tatari-cymosum*, *F. megaspartanium*, *F. pilus*, and *F. cymosum* was 13.12, 8.65, 7.21, 5.54, 6.07, and 6.56 g·kg^-1^, respectively. The average phytolith content of these six *Fagopyrum* species studied was 13.26, 6.52, 4.24, 1.96, 2.20, and 2.63 g·kg^-1^, respectively. The average PhytOC content of these six *Fagopyrum* species was 0.38, 0.16, 0.14, 0.07, 0.06, and 0.07 g·kg^-1^, respectively. There were significant differences in total Si, phytolith, and PhytOC content between these six different *Fagopyrum* species (**Figure 2**). Total Si, phytolith, and PhytOC contents of *F. esculentum* were significantly higher than those of the remaining five species. In addition, there was no significant difference in total Si, phytolith, and PhytOC content among these remaining five *Fagopyrum* species.

#### Total silicon, phytolith, and phytolith-occluded organic carbon contents between organs

**Figure 3:**
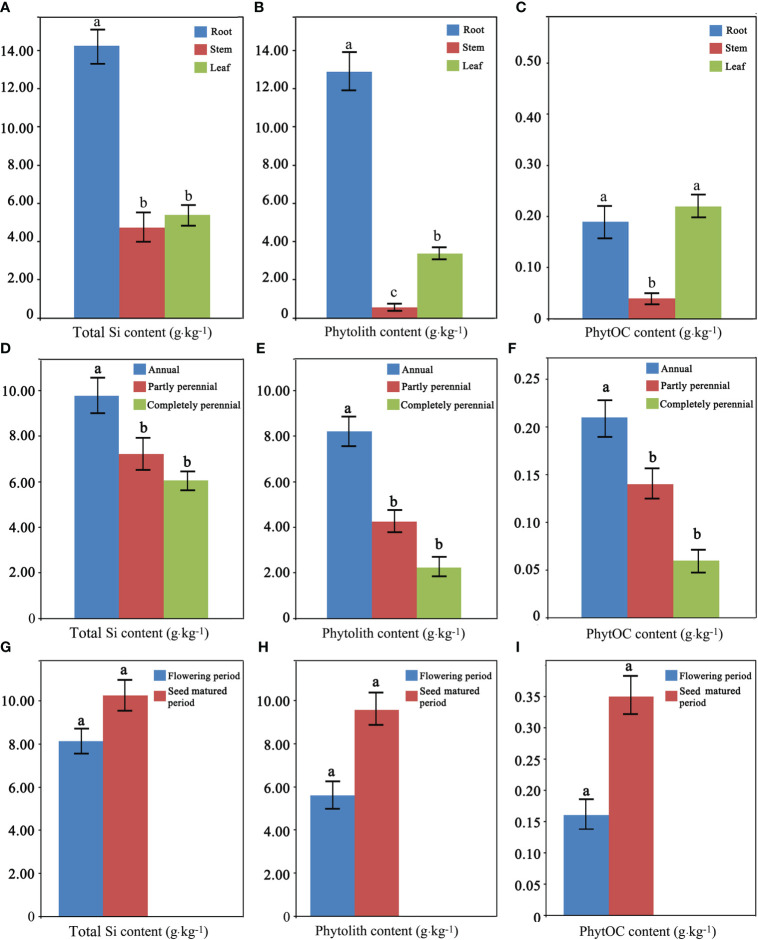
Total silicon (Si) **(A, D**, **G)**, phytolith **(B, E**, **H)**, and phytolith-occluded organic carbon (PhytOC) **(C, F**, **I)** content between different organs **(A–C)**, different periods **(D–F)**, and different live forms **(G–I)** of *Fagopyrum* plants.

The average total Si content of the root, stem, and leaf of the whole 17 *Fagopyrum* plants studied was 14.25, 4.73, and 5.41 g·kg^-1^, respectively. The average phytolith content of the root, stem, and leaf was 12.89, 0.55, and 3.36 g·kg^-1^, respectively. The average PhytOC content of the root, stem, and leaf was 0.19, 0.04, and 0.22 g·kg^-1^, respectively. There were significant differences in total Si, phytolith, and PhytOC content between the three different organs of the whole 17 *Fagopyrum* plants studied (**Figures 3A–C**). The total Si and phytolith contents of roots were the highest, significantly higher than those of stems and leaves. There was no difference in the total Si content between stems and leaves, but the phytolith content of leaves was significantly higher than that of stems. The PhytOC content of roots and leaves was significantly higher than that of stems.

#### Total silicon, phytolith, and phytolith-occluded organic carbon contents between living forms

The average total Si content of the three living forms, annual, partly perennial, and completely perennial types, of the whole 17 *Fagopyrum* plants studied was 9.77, 7.21, and 6.06 g·kg^-1^, respectively. The average phytolith content of the three living form plants was 8.21, 4.24, and 2.24 g·kg^-1^, respectively. The average PhytOC content of the three living form plants was 0.21, 0.14, and 0.06 g·kg^-1^, respectively. There were significant differences in the total Si, phytolith, and PhytOC content between the three different living form plants studied (**Figures 3D–F**). The total Si, phytolith, and PhytOC contents of annual plants were significantly higher than those of partly perennial and completely perennial plants. In addition, there was no significant difference in the total Si, phytolith, and PhytOC content between partly perennial and completely perennial plants.

#### Total silicon, phytolith, and phytolith-occluded organic carbon contents between growth periods

The average total Si content of *Fagopyrum* plants in the flowering (May) and seed matured (July) period was 8.13 and 10.26 g·kg^-1^, respectively. The average phytolith content of the flowering and seed-matured period plants was 5.60 and 9.58 g·kg^-1^, respectively. The average PhytOC content of the flowering and seed-matured period plants was 0.16 and 0.35 g·kg^-1^, respectively. There was no significant difference in the total Si, phytolith, and PhytOC content of *Fagopyrum* plants between these two growth periods (**Figures 3G–I** **)**.

### Phytolith carbon sink potential of cultivated buckwheat planting

#### Phytolith carbon sequestration rate of *Fagopyrum esculentum* and *Fagopyrum tataricum planting*

The average dry biomass of *F. esculentum* and *F. tataricum* individuals was 2.65 g and 2.73 g, respectively (**Table 2**). Based on the average PhytOC content, the average PhytOC reserve of *F. esculentum* and *F. tataricum* individuals was calculated and the value was 5.95 × 10^-4^ g and 2.65 × 10^-4^ g, respectively. When the planting frequentness was set by one time per year, the phytolith carbon sequestration rate of *F. esculentum* and *F. tataricum* planting was 2.62 × 10^-3^ and 1.17 × 10^-3^ t CO_2_ hm^-2^·a^-1^, respectively.

**Table 2:** PhytOC reserves of individual plant and phytolith carbon sequestration rate of *F. esculentum* and *F. tataricum* planting.

Species	Biomass dry weight and PhytOC reserves of individual plant (g)								Phytolith carbon sequestration rate (phytolith carbon sequestration amount per hectare and year)			
	Root		Stem		Leaf		Total		Planting density (individuals·hm^-2^)	Biomass dry weight of one hectare(kg)	Phytolith carbon sequestration amount per hectare(kg)	Phytolith carbon sequestration amount per hectare and year(t CO_2_ hm^-2^·a^-1^)
	Biomass dry weight	PhytOC reserves	Biomass dry weight	PhytOC reserves	Biomass dry weight	PhytOC reserves	Biomass dry weight	PhytOC reserves			
*F. esculentum*	0.42	2.18×10^-4^	1.81	1.36×10^-4^	0.43	2.42×10^-4^	2.65	5.95×10^-4^	120×10^4^	3182.4	0.71	2.62×10^-3^
*F. tataricum*	0.43	0.91×10^-4^	1.88	0.77×10^-4^	0.42	0.97×10^-4^	2.73	2.65×10^-4^	120×10^4^	3273.6	0.32	1.17×10^-3^
Average	0.42	1.55×10^-4^	1.85	1.07×10^-4^	0.42	1.69×10^-4^	2.69	4.30×10^-4^	120×10^4^	3228.0	0.52	1.89×10^-3^

PhytOC, phytolith-occluded organic carbon.

#### Annual total amount of phytolith carbon sequestration of Chinese buckwheat planting

**Figure 4:**
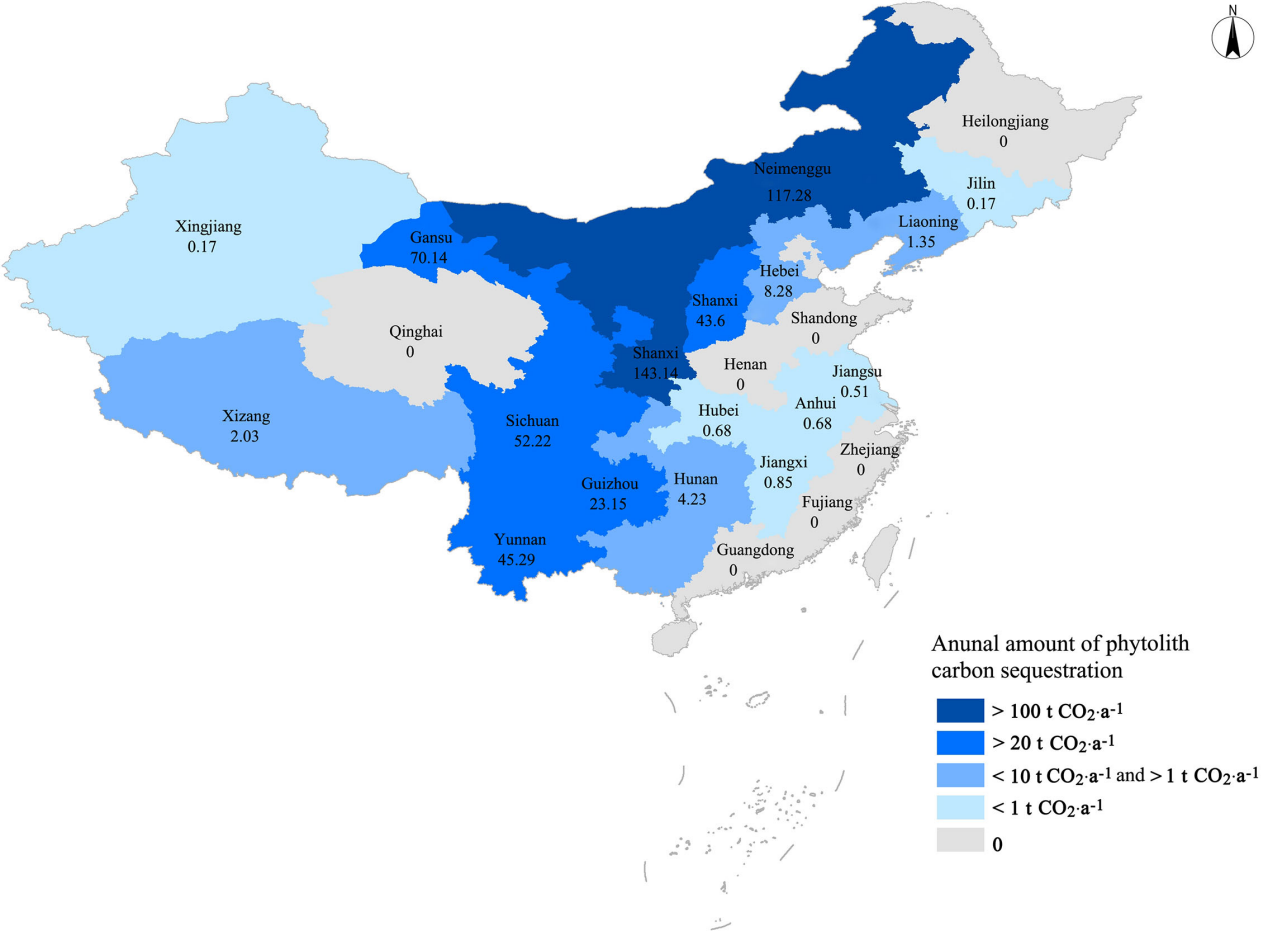
Annual amount and distribution of phytolith carbon sequestration of Chinese buckwheat planting in 2020.

The data of the National Statistics Bureau of China (https://data.stats.gov.cn/) and United Nations Food and Agriculture Organization (http://fao.org./home/zh) did not provide the specific area of *F. esculentum* and *F. tataricum* planting. The average phytolith carbon sequestration rate of *F. esculentum* and *F. tataricum* planting, 1.89 × 10^-3^ t CO_2_ hm^-2^·a^-1^ (**Table 2**), was used to calculate the annual total amount of phytolith carbon sequestration. In 2020, the total planting area of cultivated buckwheat was 369,700 hectares and the annual total amount of phytolith carbon sequestration of buckwheat planting was 624.79 t CO_2_ in China, mainly in Shaanxi, Inner Mongolia, Ningxia, Gansu, Sichuan, Yunnan, Shanxi, and Guizhou (**Figure 4**).

#### Annual total amount of phytolith carbon sequestration of global buckwheat planting

**Figure 5:**
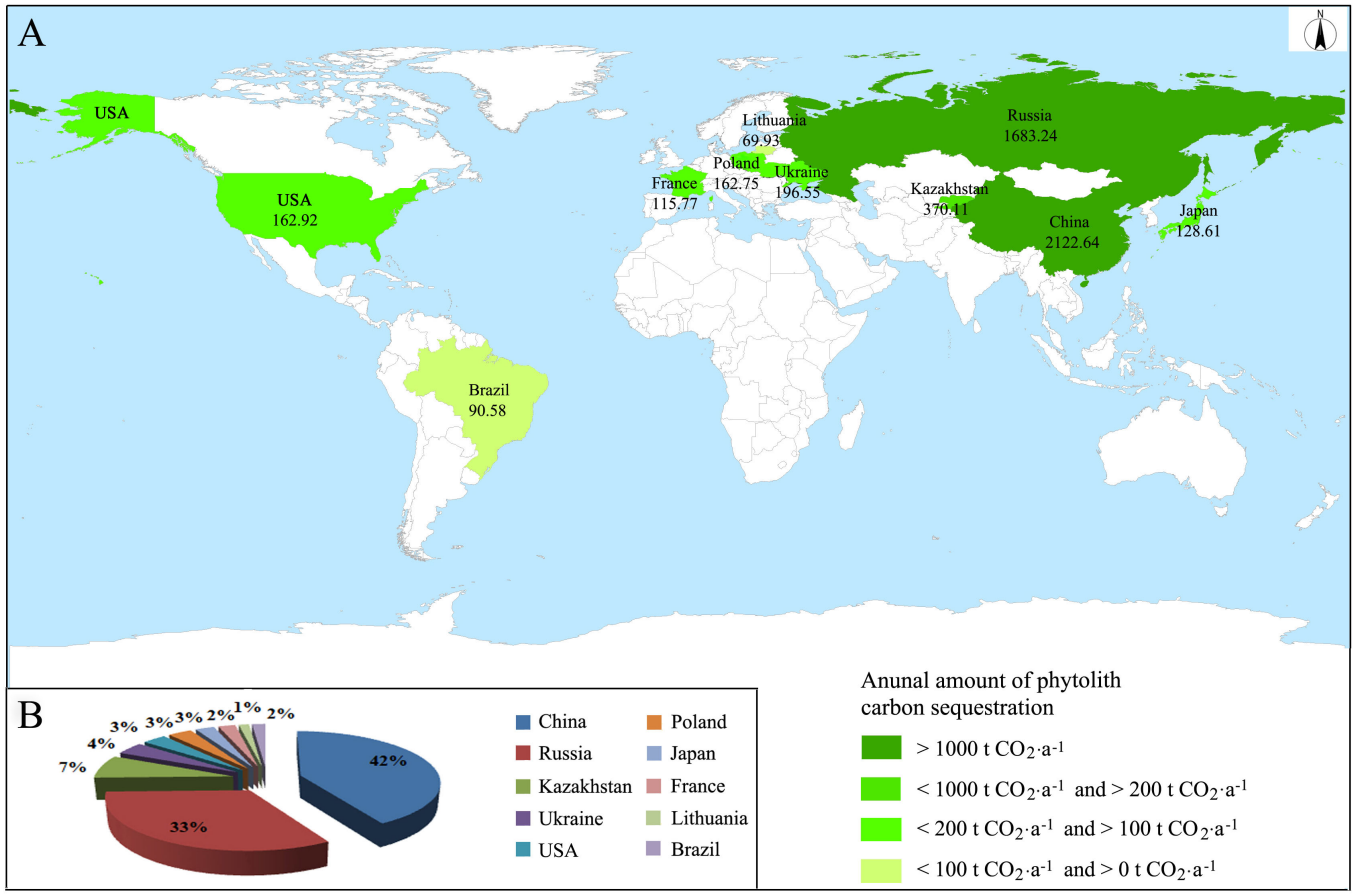
Annual amount and distribution of phytolith carbon sequestration of global buckwheat planting in 2018. **(A)** Distribution, **(B)** Proportions.

In 2018, the total planting area of cultivated buckwheat was 3,019,400 hectares and the annual total amount of phytolith carbon sequestration of buckwheat planting was 5,102.79 t CO_2_ in the whole world, mainly in China, Russia, Kazakhstan, Ukraine, United States, Poland, Japan, France, Lithuania, and Brazil (**Figure 5**). The total amount of phytolith carbon sequestration of China and Russia accounted for 41.59% and 32.98% of that of the whole world, respectively. In addition, the sum of the total amount of phytolith carbon sequestration of China and Russia accounted for more than 70% of that of the whole world.

## Discussion

### Phytolith-occluded organic carbon content in *Fagopyrum* plants

In herbaceous crops, plants of the grass family have a high PhytOC content (Parr et al., 2010; Li et al., 2014; Nguyen et al., 2014). The average PhytOC content of rice and sugarcane reached 0.25% (Parr et al., 2009). The average PhytOC content of wheat, corn, and cereal was 0.16%, 0.16%, and 0.17%, respectively (Song et al., 2013). The PhytOC contents of bean, potato, and oil crops also have been reported, with values of 0.02%, 0.08%, and 0.02%, respectively (Zuo and Lü, 2011). However, until now, there still is no report on buckwheat phytolith and PhytOC studies. Compared with other crops reported (Parr and Sullivan, 2011; Zuo and Lü, 2011; Guo et al., 2015), the PhytOC content of buckwheat is remarkably less than that of rice, wheat, corn, cereal, sugarcane, and oil crops. Among these six buckwheat species, the PhytOC content of *F. esculentum* (0.038%) was higher than that of bean, potato, and cotton (Parr and Sullivan, 2011; Zuo and Lü, 2011); the PhytOC content of *F. tataricum* and *F. tatari-cymosum* (0.016% and 0.014%, respectively) was close to that of bean, potato, and cotton (Guo et al., 2015). In addition, the PhytOC content of the three wild species of *F. megaspartanium*, *F. pilus*, and *F. cymosum* (0.007%, 0.006%, and 0.007%, respectively) was significantly less than that of beans, potato, and cotton (Guo et al., 2015).

The ability to absorb soil Si between different plants is obviously different (Ma, 2003), so that plant genetic properties have significant impacts on their PhytOC accumulation (Zuo and Lü, 2011). A large number of studies have confirmed that significant differences were present in the PhytOC content between different intergeneric species (Ma, 2003; Ray et al., 2004), but there still is no systematic report on whether the PhytOC content varied significantly between intragenus species. The present study results showed, among the six *Fagopyrum* species, that the total Si, phytolith, and PhytOC contents of *F. tataricum* were significantly higher than those of the remaining five species, showing that there were significant differences in total Si, phytolith, and PhytOC content between different intragenus species of *Fagopyrum*. Phytoliths in plants mainly distribute in epidermal cells of the roots, stems, and leaves, whereas phytolith and PhytOC contents in different organs are obviously different (Ray et al., 2004; Zhan et al., 2019; Zhong et al., 2020). Studies on rice phytolith contents showed that there were significant differences in the PhytOC content between different organs sheath, leaf, stem, root, and ear, and the PhytOC content of the sheath and leaf was significantly higher than that of the stem, root, and ear (Schaller et al., 2019). The present study obtained a consistent result with the above study and showed that there were significant differences in the buckwheat PhytOC content between different organs root, stem, and leaf. In addition, the PhytOC content of the leaf was significantly higher than that of the stem in buckwheat plants. The phytolith content in plant tissues could be affected by seasonal changes, and the content reached its highest in July and then gradually decreased (Zhao et al., 2015; Yang et al., 2019). The present results showed that there was no significant difference in the total Si, phytolith, and PhytOC content between May and July, which can be attributed to the fact that the time interval of these two periods of buckwheat was very short and this short time interval did not result in a significant change in PhytOC accumulation in buckwheat plants. Moreover, the present study showed that there were significant differences in the total Si, phytolith, and PhytOC content between different living forms of buckwheat plants. However, different from expectations, the total Si, phytolith, and PhytOC contents of annual plants were significantly higher than those of partly perennial and completely perennial plants. In the present study, the two annual plants, *F. esculentum* and *F. tataricum*, are cultivated species and the partly perennial and completely perennial plants are wild species. Cultivated plants are bred for high biomasses and received high loads of nutrients by fertilization. Therefore, it can be inferred that cultivated species have a more rapid growth speed than wild species, which is helpful to the accumulation of the total Si, phytolith, and PhytOC and results in cultivated species having a higher PhytOC content than that in wild species within the same genus.

The plant PhytOC content is closely related to phytolith content, carbon fixation efficiency of phytoliths, and its growth environments (Ying et al., 2016; Zhang et al., 2020). Consistent with expectations, the present results showed that there was an extremely significant positive correlation between the PhytOC and phytolith content and between the phytolith and total Si content (**Table 3**). Soil is the supporter of plant growth. Soil physical and chemical environmental conditions obviously affect plant growth (Sheng et al., 2018), and the Si element in plants comes from soils. Therefore, it can be assumed that soil physical and chemical properties can obviously affect plant PhytOC content. However, the present results showed that the total Si, phytolith, and PhytOC content of plants had no significant correlation with soil physical and chemical properties (**Table 4**). In the present study, the buckwheat materials studied were planted in the karst area of Southwest China. Yang et al. (2021) found that the plant-available Si was abundant in Southwest China. Therefore, it can be inferred that in Southwest China, although Si is a necessary element for plant phytolith production and soil is the only source of plant Si element, the plant-available Si in soils is rich and enough to ensure the demands of plant growth and phytolith production, leading to that the changes in soil physical and chemical properties did not cause the obvious variation of the total Si, phytolith, and PhytOC content in plants. This conclusion also was supported by a study by Li et al. (2021). The conclusions of Li et al. (2021) showed that the abundant Si element of karst rocks resulted in the high plant-available Si content of karst soils in Southwest China.

**Table 3:** Correlations among total Si, phytolith, and PhytOC contents of *Fagopyrum* plants.

	Total Si content	Phytolith content	PhytOC content
Total Si content	1	0.868**	-0.002
Phytolith content	0.927**	1	0.307**
PhytOC content	0.831**	0.740**	1

The lower left of the table shows the Pearson correlation results between pairwise variables. The upper right of the table shows partial correlation analysis results.

* and ** indicate significant correlation (α = 0.05) and extremely significant correlation (α = 0.01), respectively.

PhytOC, phytolith-occluded organic carbon; Si, silicon.

**Table 4:** Correlations between total Si, phytolith, and PhytOC content of plants and physicochemical factors of sample plot and rhizosphere soils.

Factors		*Fagopyrum* plants		
		Total Si content	Phytolith content	PhytOC content
Sample plot soils	BD	0.169	0.184	0.102
	NWC	-0.009	0.030	0.130
	FMC	-0.429	-0.372	-0.310
	pH	-0.172	-0.201	-0.270
	TP	-0.008	0.047	0.063
	TSOC	-0.008	0.047	0.063
	TN	-0.008	0.047	0.063
	Available Si	-0.170	-0.160	-0.113
Rhizosphere soils	pH	-0.309	-0.343	-0.403
	TP	0.706	0.752	0.749
	TSOC	-0.204	-0.211	-0.092
	TN	0.376	0.344	0.388
	Available Si	0.101	0.117	0.052

BD, bulk density; NWC, natural water content; FMC, field moisture capacity; TP, total phosphorus; TSOC, total soil organic carbon; TN, total nitrogen; PhytOC, phytolith-occluded organic carbon; Si, silicon.

### Phytolith carbon sink potential of cultivated buckwheat planting

Although it has been a consensus that PhytOC is very difficult to decompose under the protection of extremely stable phytoliths (Yang et al., 2016; de Tombeur et al., 2021) and most phytoliths and PhytOC can be preserved in soils after plants are rotten and burned completely (Parr and Sullivan, 2005), there are still some controversies in plant phytolith carbon sink potential that has been hypothesized to be quite high or low (Hodson, 2019). The present results showed that although the phytolith and PhytOC content of buckwheat was less than those of the grass family crops rice, wheat, and corn, buckwheat, as a non-grass family herbaceous crop, still had an obvious potential of phytolith carbon sink. Although it is not absolutely confirmed that the PhytOC content measured by the present method really occluded wholly in phytoliths and there may be organic contaminants on the surface of extracted phytoliths, it still can be concluded that herbaceous crops widely planted in the whole world have an important phytolith carbon sink function and the obvious potential of playing important roles in carbon cycle regulation (Song et al., 2014; Nguyen et al., 2019).

The phytolith and PhytOC contents of different crop plants are obviously different, resulting in that there are remarkable differences in phytolith carbon sequestration rate and phytolith carbon sink potential between different crops. The phytolith carbon sequestration rate of grass family crops is high (Song et al., 2014). In herbaceous crops, rice has the highest phytolith carbon sequestration rate, up to 0.0678 t CO_2_ hm^-2^·a^-1^ (Li et al., 2013). Wheat and corn also have higher phytolith carbon sequestration rates, with values of 0.0375 t CO_2_ hm^-2^·a^-1^ (Parr and Sullivan, 2011) and 0.0444 t CO_2_ hm^-2^·a^-1^ (Song et al., 2014), respectively. The present results showed that the average phytolith carbon sequestration rate of *F. esculentum* and *F. tataricum* was 2.62 × 10^-3^ and 1.17 × 10^-3^ t CO_2_ hm^-2^·a^-1^, respectively, significantly less than that of the above crops. In general, the phytolith content and carbon sink potential of plants are closely related to their self-biomass (Wehrhan et al., 2021). Therefore, crops with large biomass have an obviously big capacity for phytolith carbon sink. Because of the large biomass, the annual total amount of phytolith carbon sequestration of sugarcane reaches 0.096 t CO_2_ hm^-2^·a^-1^ (Parr et al., 2009). In addition, the annual total amount of phytolith carbon sequestration of millet and broomcorn millet is 0.023 and 0.02 t CO_2_ hm^-2^·a^-1^, respectively (Zuo and Lü, 2011). Due to the obvious differences in biomass, the differences in phytolith carbon sequestration capacity were more significant than those of the phytolith and PhytOC content between buckwheat and these above three crops. Although the phytolith and PhytOC content of buckwheat is near that of cotton (Song et al., 2014), the biomass of buckwheat is significantly less than that of cotton, with the result that the phytolith carbon sequestration capacity of buckwheat is significantly less than that of cotton.

Compared with the phytolith carbon sequestration rate of terrestrial natural ecosystems (Ying et al., 2016; Yang et al., 2018; Song et al., 2022), the phytolith carbon sequestration rate of buckwheat is near that of shrub vegetation (Pan et al., 2017; Ru et al., 2018). It can be seen that although the phytolith carbon sequestration rate of buckwheat is small, the phytolith carbon sink potential of buckwheat still has obvious values in the vegetation carbon sink. The present results showed that the annual total amount of phytolith carbon sequestration of Chinese buckwheat planting reached 624.79 t CO_2_ in 2020 and that of global buckwheat planting reached 5,102.79 t CO_2_ in 2018. China is the main distribution area of *Fagopyrum* plants and the largest buckwheat planting country in the whole world (Sheng et al., 2013). In addition, the annual total amount of phytolith carbon sequestration of Chinese buckwheat planting accounts for more than 40% of that of the whole world. Thus, it can be concluded that although the carbon sequestration rate of buckwheat phytoliths is low, the planting area in China is very large and PhytOC is extremely stable and can accumulate for a very long time, with the result that the phytolith carbon sink of buckwheat planting has the obvious potential of playing an important role in carbon cycle regulation and Chinese carbon neutral strategy. However, until now, there still is no standard protocol for the determination of PhytOC. To accurately estimate the potential of the phytolith carbon sink, a lot of work on building a standard protocol for the determination of PhytOC is urgent and of significance.

## Conclusions

The PhytOC content of the two cultivated buckwheat species *F. esculentum* and *F. tataricum* was near that of bean, potato, and cotton. The total Si, phytolith, and PhytOC content of *F. esculentum* was the highest among the six species studied. In addition, the total Si, phytolith, and PhytOC content of annual *Fagopyrum* plants was significantly higher than those of partly and completely perennial *Fagopyrum* plants. There was an extremely significant positive correlation between the plant PhytOC and phytolith content and between the plant phytolith and total Si content. The abundant soil plant-available Si resulted in that the total Si, phytolith, and PhytOC content of *Fagopyrum* plants had no significant correlation with soil physical and chemical properties in the Southwest China karst area. The average of phytolith carbon sequestration rate of *F. esculentum* and *F. tataricum* planting is approximately equal to that of the terrestrial shrub natural ecosystem. The annual total amount of phytolith carbon sequestration of Chinese-cultivated buckwheat planting reached 624.79 t CO_2_ in 2020 and that of global cultivated buckwheat planting was 5,102.79 t CO_2_ in 2018. The buckwheat phytolith carbon sink has the obvious potential of playing important roles in carbon cycle regulation.

In the present study, the buckwheat phytolith carbon sink was estimated based on single-region data. Different regional climates and soil conditions can obviously affect the PhytOC accumulation in plants. Thus, the present estimation of buckwheat phytolith carbon sink needs to become more precise. In future research, adding more samples from the whole world to more accurately calculate the carbon sink is very much needed. Until now, there still is no standard protocol for the determination of PhytOC. To accurately estimate the potential of the phytolith carbon sink, studies on building a standard protocol for the determination of PhytOC are urgent and of significance.

## Data availability statement

The original contributions presented in the study are included in the article/**Supplementary Material**. Further inquiries can be directed to the corresponding author.

## Author contributions

LW participated in the design of the study, carried out the fieldwork, collected primary data and mined data, carried out the statistical analyses, and drafted the manuscript. MS designed the study and critically revised the manuscript. All authors gave final approval for publication and agreed to be held accountable for the work performed therein.

## Funding

This work was supported by grants from the China National Natural Science Foundation (42107250), and the Key Project of Guizhou Science and Technology Fund (Qiankehe Jichu [2020]1Z012).

## Acknowledgments

We thank Yaqi Zhou, Jie Yin, Faying Yuan, Suili Zhang, and Xianxian He for assistance with collecting plant and soil samples.

## Conflict of interest

The authors declare that the research was conducted in the absence of any commercial or financial relationships that could be construed as a potential conflict of interest.

## Publisher’s note

All claims expressed in this article are solely those of the authors and do not necessarily represent those of their affiliated organizations, or those of the publisher, the editors and the reviewers. Any product that may be evaluated in this article, or claim that may be made by its manufacturer, is not guaranteed or endorsed by the publisher.
