# Supplementary material for: Phytolith occluded organic carbon in Fagopyrum (Polygonaceae) plants: Insights on the carbon sink potential of cultivated buckwheat planting
Source: Front Plant Sci. 2022 Nov 11;13:1014980. doi: 10.3389/fpls.2022.1014980 (PMC9692092; doi:10.3389/fpls.2022.1014980)
Supplement: Supplementary file 1 [file DataSheet_1.docx]

**Supplementary data**

**Manuscript title:**

Phytolith occluded organic carbon in *Fagopyrum* (Polygonaceae) plants: insights for carbon sink potential of buckwheat planting

**Authors:**

Linjiao Wang, Maoyin Sheng^*^

**Contents:**

**Supplementary table S1**: Soil physical and chemical properties of sample plots

**Supplementary table S2**: pH, TP, TN, TSOC and Asi content of sample rhizosphere soils

**Supplementary table S3**: Total Si content of *Fagopyrum* plants studied

**Supplementary table S4**: Phytolith content of *Fagopyrum* plants studied

**Supplementary table S5**: PhytOC content of *Fagopyrum* plants studied

**Supplementary table S1**: Soil physical and chemical properties of sample plots

| *Fagopyrum*  plants | Soil layer | BD  (g·cm^-3^) | NWC  (%) | FWC  (%) | pH | | | TP  (g·kg^-1^) | | | TSOC  (g·kg^-1^) | | | TN  (g·kg^-1^) | | | ASi  (mg·kg^-1^) | | |
| --- | --- | --- | --- | --- | --- | --- | --- | --- | --- | --- | --- | --- | --- | --- | --- | --- | --- | --- | --- |
|  |  |  |  |  | 1 | 2 | 3 | 1 | 2 | 3 | 1 | 2 | 3 | 1 | 2 | 3 | 1 | 2 | 3 |
| Hongtian 5# | 0-5 cm | 1.15 | 32.01 | 30.88 | 5.80 | 5.78 | 5.81 | 0.81 | 0.78 | 0.79 | 23.93 | 26.17 | 24.84 | 0.11 | 0.11 | 0.11 | 77.60 | 76.54 | 76.23 |
|  | 5-10 cm | 1.27 | 41.42 | 33.05 | 5.75 | 5.76 | 5.74 | 0.93 | 0.92 | 0.87 | 23.59 | 23.55 | 24.25 | 0.11 | 0.11 | 0.11 | 74.13 | 74.86 | 72.50 |
|  | 10-20 cm | 1.30 | 40.05 | 40.09 | 5.61 | 5.64 | 5.63 | 0.72 | 0.75 | 0.73 | 23.55 | 23.25 | 23.89 | 0.10 | 0.09 | 0.11 | 83.96 | 80.97 | 82.48 |
| Hongtian 2# | 0-5 cm | 1.20 | 27.20 | 25.54 | 5.78 | 5.75 | 5.76 | 1.04 | 0.91 | 0.97 | 19.39 | 18.12 | 19.05 | 0.10 | 0.09 | 0.07 | 74.59 | 77.73 | 74.36 |
|  | 5-10 cm | 1.17 | 30.74 | 27.41 | 5.66 | 5.69 | 5.66 | 0.81 | 0.81 | 0.86 | 17.43 | 18.11 | 17.47 | 0.08 | 0.08 | 0.16 | 71.32 | 73.04 | 70.95 |
|  | 10-20 cm | 1.31 | 30.97 | 28.62 | 5.49 | 5.51 | 5.51 | 0.77 | 0.80 | 0.22 | 16.48 | 17.13 | 17.13 | 0.07 | 0.07 | 0.09 | 88.36 | 77.52 | 81.16 |
| Heimi-15 | 0-5 cm | 1.23 | 30.05 | 30.18 | 5.94 | 5.87 | 5.96 | 0.66 | 0.66 | 0.66 | 18.09 | 17.46 | 18.40 | 0.09 | 0.09 | 0.10 | 71.61 | 70.81 | 75.42 |
|  | 5-10 cm | 1.60 | 31.62 | 29.53 | 5.85 | 5.84 | 5.83 | 0.73 | 0.73 | 0.69 | 18.72 | 18.45 | 18.43 | 0.10 | 0.09 | 0.08 | 96.34 | 96.86 | 93.59 |
|  | 10-20 cm | 1.47 | 32.26 | 31.19 | 5.85 | 5.77 | 5.72 | 0.68 | 0.69 | 0.71 | 17.13 | 17.76 | 17.74 | 0.09 | 0.09 | 0.09 | 105.98 | 102.34 | 100.41 |
| Yunku 1# | 0-5 cm | 1.22 | 27.12 | 36.03 | 6.07 | 6.05 | 6.05 | 0.97 | 0.96 | 0.96 | 16.78 | 16.79 | 17.48 | 0.09 | 0.08 | 0.09 | 125.65 | 124.51 | 125.79 |
|  | 5-10 cm | 1.31 | 26.83 | 35.67 | 6.03 | 6.06 | 6.03 | 0.92 | 0.94 | 0.93 | 15.83 | 16.18 | 15.83 | 0.09 | 0.09 | 0.10 | 109.91 | 103.53 | 106.42 |
|  | 10-20 cm | 1.35 | 31.10 | 29.85 | 6.04 | 6.05 | 6.03 | 0.79 | 0.83 | 0.78 | 13.87 | 14.54 | 13.88 | 0.08 | 0.08 | 0.09 | 97.19 | 95.23 | 91.91 |
| Kuqiao YZX | 0-5 cm | 1.21 | 27.15 | 37.13 | 6.05 | 6.07 | 6.05 | 0.94 | 0.98 | 0.90 | 16.48 | 17.45 | 17.44 | 0.09 | 0.10 | 0.08 | 64.91 | 69.39 | 75.37 |
|  | 5-10 cm | 1.15 | 31.20 | 30.34 | 6.06 | 6.04 | 6.05 | 0.92 | 0.98 | 0.94 | 12.91 | 13.57 | 12.90 | 0.08 | 0.09 | 0.09 | 66.36 | 66.37 | 61.43 |
|  | 10-20 cm | 1.18 | 32.25 | 37.03 | 6.01 | 6.01 | 6.02 | 0.85 | 0.87 | 0.86 | 16.14 | 16.48 | 16.44 | 0.08 | 0.08 | 0.09 | 78.84 | 86.59 | 73.72 |

**Supplementary table S1** (Continual)

| *Fagopyrum*  plants | Soil layer | BD  (g·cm^-3^) | NWC  (%) | FWC  (%) | pH | | | TP  (g·kg^-1^) | | | TSOC  (g·kg^-1^) | | | TN  (g·kg^-1^) | | | ASi  (mg·kg^-1^) | | |
| --- | --- | --- | --- | --- | --- | --- | --- | --- | --- | --- | --- | --- | --- | --- | --- | --- | --- | --- | --- |
|  |  |  |  |  | 1 | 2 | 3 | 1 | 2 | 3 | 1 | 2 | 3 | 1 | 2 | 3 | 1 | 2 | 3 |
| GJK  2012-2294# | 0-5 cm | 1.06 | 33.86 | 45.26 | 5.78 | 5.76 | 5.78 | 0.96 | 0.98 | 0.89 | 22.96 | 22.93 | 22.30 | 0.11 | 0.23 | 0.11 | 96.96 | 94.83 | 97.43 |
|  | 5-10 cm | 1.33 | 36.15 | 38.30 | 5.76 | 5.75 | 5.75 | 0.95 | 1.01 | 0.97 | 24.91 | 24.60 | 25.20 | 0.10 | 0.10 | 0.11 | 103.31 | 101.25 | 103.39 |
|  | 10-20 cm | 1.21 | 32.94 | 38.62 | 5.68 | 5.65 | 5.66 | 0.84 | 0.88 | 0.83 | 22.01 | 22.65 | 22.58 | 0.10 | 0.10 | 0.09 | 104.31 | 101.37 | 98.19 |
| GJK  2012-2298# | 0-5 cm | 1.01 | 33.56 | 47.49 | 5.81 | 5.86 | 5.81 | 0.92 | 0.91 | 0.94 | 20.35 | 20.08 | 19.40 | 0.10 | 0.10 | 0.09 | 86.54 | 88.72 | 89.75 |
|  | 5-10 cm | 1.08 | 35.24 | 34.96 | 5.76 | 5.75 | 5.75 | 0.84 | 0.86 | 0.87 | 22.34 | 21.99 | 22.25 | 0.10 | 0.09 | 0.10 | 84.83 | 88.71 | 85.43 |
|  | 10-20 cm | 1.09 | 35.18 | 34.14 | 5.73 | 5.74 | 5.75 | 0.79 | 0.82 | 0.83 | 19.09 | 18.40 | 18.38 | 0.10 | 0.08 | 0.08 | 90.08 | 95.97 | 94.42 |
| GJK  2012-1206# | 0-5 cm | 1.00 | 1.53 | 41.18 | 6.11 | 6.10 | 6.11 | 0.84 | 0.80 | 0.85 | 18.38 | 18.72 | 18.09 | 0.13 | 0.13 | 0.12 | 130.39 | 131.26 | 130.22 |
|  | 5-10 cm | 1.03 | 39.43 | 50.81 | 6.08 | 6.11 | 6.09 | 0.83 | 0.83 | 0.80 | 17.43 | 16.48 | 17.12 | 0.10 | 0.09 | 0.09 | 96.39 | 97.90 | 95.90 |
|  | 10-20 cm | 1.02 | 38.30 | 36.86 | 6.01 | 6.02 | 6.02 | 0.77 | 0.73 | 0.75 | 16.12 | 16.77 | 16.18 | 0.08 | 0.09 | 0.09 | 113.51 | 109.74 | 107.40 |
| GMK  2010-2002# | 0-5 cm | 1.29 | 27.45 | 43.71 | 5.67 | 5.65 | 5.67 | 1.01 | 1.04 | 1.03 | 11.32 | 11.94 | 11.93 | 0.06 | 0.07 | 0.06 | 69.96 | 67.21 | 67.08 |
|  | 5-10 cm | 1.37 | 2.34 | 31.28 | 5.58 | 5.62 | 5.54 | 0.91 | 0.91 | 0.95 | 12.93 | 12.93 | 13.55 | 0.06 | 0.07 | 0.06 | 71.73 | 73.70 | 79.06 |
|  | 10-20 cm | 1.54 | 30.35 | 36.70 | 5.51 | 5.50 | 5.49 | 0.87 | 0.81 | 0.87 | 12.93 | 13.89 | 13.26 | 0.05 | 0.05 | 0.06 | 88.57 | 73.74 | 81.14 |
| GMK  2012-2163# | 0-5 cm | 1.22 | 25.67 | 38.87 | 5.89 | 5.90 | 5.90 | 0.96 | 0.94 | 0.93 | 15.15 | 15.82 | 15.48 | 0.07 | 0.07 | 0.08 | 62.49 | 63.45 | 70.37 |
|  | 5-10 cm | 1.26 | 25.88 | 36.57 | 6.84 | 6.86 | 6.86 | 0.88 | 0.92 | 0.93 | 15.80 | 16.49 | 16.80 | 0.08 | 0.07 | 0.07 | 56.68 | 42.81 | 45.41 |
|  | 10-20 cm | 1.31 | 28.93 | 32.93 | 5.79 | 5.82 | 5.86 | 0.85 | 0.83 | 0.81 | 17.77 | 18.10 | 18.14 | 0.07 | 0.08 | 0.08 | 53.47 | 54.73 | 59.54 |

**Supplementary table S1** (Continual)

| *Fagopyrum*  plants | Soil layer | BD  (g·cm^-3^) | NWC  (%) | FWC  (%) | pH | | | TP  (g·kg^-1^) | | | TSOC  (g·kg^-1^) | | | TN  (g·kg^-1^) | | | ASi  (mg·kg^-1^) | | |
| --- | --- | --- | --- | --- | --- | --- | --- | --- | --- | --- | --- | --- | --- | --- | --- | --- | --- | --- | --- |
|  |  |  |  |  | 1 | 2 | 3 | 1 | 2 | 3 | 1 | 2 | 3 | 1 | 2 | 3 | 1 | 2 | 3 |
| GMK  2012-19# | 0-5 cm | 1.33 | 25.23 | 39.70 | 5.79 | 5.77 | 5.77 | 1.03 | 0.96 | 0.98 | 17.44 | 18.12 | 17.11 | 0.07 | 0.07 | 0.07 | 59.69 | 57.24 | 57.73 |
|  | 5-10 cm | 1.17 | 24.51 | 40.64 | 5.74 | 5.78 | 5.78 | 0.88 | 0.85 | 0.85 | 17.81 | 17.11 | 17.73 | 0.08 | 0.08 | 0.07 | 54.85 | 53.42 | 53.86 |
|  | 10-20 cm | 1.29 | 26.63 | 36.72 | 5.75 | 5.77 | 5.75 | 0.81 | 0.81 | 0.83 | 15.15 | 15.87 | 14.88 | 0.06 | 0.07 | 0.06 | 54.19 | 52.54 | 52.84 |
| HXJQ | 0-5 cm | 1.16 | 35.73 | 43.62 | 5.75 | 5.69 | 5.72 | 1.07 | 1.04 | 1.02 | 23.54 | 23.87 | 23.29 | 0.13 | 0.11 | 0.10 | 73.97 | 70.33 | 68.31 |
|  | 5-10 cm | 1.20 | 37.49 | 43.17 | 5.71 | 5.68 | 5.70 | 0.84 | 0.81 | 0.82 | 23.56 | 23.91 | 22.96 | 0.13 | 0.09 | 0.11 | 73.03 | 73.62 | 73.24 |
|  | 10-20 cm | 1.35 | 36.18 | 35.19 | 5.63 | 5.63 | 5.65 | 0.74 | 0.74 | 0.71 | 24.58 | 24.18 | 23.57 | 0.10 | 0.09 | 0.11 | 87.32 | 77.30 | 74.50 |
| JQ 1# | 0-5 cm | 1.21 | 35.83 | 46.28 | 6.04 | 6.02 | 6.03 | 0.77 | 0.80 | 0.77 | 22.60 | 20.68 | 21.61 | 0.10 | 0.11 | 0.11 | 124.45 | 131.76 | 120.81 |
|  | 5-10 cm | 1.16 | 41.48 | 46.69 | 5.90 | 5.89 | 5.89 | 0.78 | 0.79 | 0.77 | 19.69 | 19.36 | 20.67 | 0.09 | 0.10 | 0.11 | 110.78 | 109.20 | 112.83 |
|  | 10-20 cm | 1.49 | 37.71 | 37.26 | 5.69 | 5.68 | 5.68 | 0.72 | 0.70 | 0.75 | 21.00 | 20.32 | 20.96 | 0.10 | 0.09 | 0.08 | 99.65 | 100.42 | 97.97 |
| JQ 3# | 0-5 cm | 1.07 | 23.89 | 25.35 | 5.57 | 5.55 | 5.56 | 0.67 | 0.65 | 0.65 | 19.35 | 20.63 | 20.64 | 0.09 | 0.08 | 0.09 | 88.12 | 85.59 | 83.97 |
|  | 5-10 cm | 1.03 | 26.17 | 26.84 | 5.54 | 5.52 | 5.52 | 0.68 | 0.65 | 0.65 | 21.64 | 21.32 | 22.30 | 0.08 | 0.08 | 0.08 | 87.14 | 84.07 | 76.03 |
|  | 10-20 cm | 1.28 | 30.07 | 30.65 | 5.41 | 5.42 | 5.41 | 0.59 | 0.60 | 0.61 | 19.08 | 19.10 | 18.74 | 0.08 | 0.08 | 0.08 | 79.49 | 87.02 | 73.71 |
| Low JQ | 0-5 cm | 1.19 | 37.00 | 43.03 | 5.78 | 5.79 | 5.79 | 0.92 | 0.93 | 0.95 | 22.94 | 22.32 | 22.95 | 0.11 | 0.10 | 0.11 | 84.23 | 81.58 | 80.88 |
|  | 5-10 cm | 1.39 | 36.23 | 36.58 | 5.70 | 5.68 | 5.72 | 0.84 | 0.87 | 0.89 | 22.61 | 22.59 | 22.28 | 0.11 | 0.10 | 0.09 | 85.54 | 77.06 | 83.05 |
|  | 10-20 cm | 1.45 | 34.31 | 34.22 | 5.66 | 5.61 | 5.64 | 0.74 | 0.72 | 0.77 | 17.45 | 16.81 | 16.16 | 0.09 | 0.09 | 0.08 | 76.33 | 84.18 | 94.56 |

**Supplementary table S1** (Continual)

| *Fagopyrum*  plants | Soil layer | BD  (g/cm^3^) | NWC  (%) | FWC  (%) | pH | | | TP  (g·kg^-1^) | | | TSOC  (g·kg^-1^) | | | TN  (g·kg^-1^) | | | ASi  (mg·kg^-1^) | | |
| --- | --- | --- | --- | --- | --- | --- | --- | --- | --- | --- | --- | --- | --- | --- | --- | --- | --- | --- | --- |
| MYQ 1# | 0-5 cm | 1.27 | 24.41 | 26.02 | 5.99 | 5.98 | 5.98 | 0.79 | 0.80 | 0.77 | 20.37 | 22.59 | 19.38 | 0.09 | 0.16 | 0.09 | 73.06 | 68.93 | 71.30 |
|  | 5-10 cm | 1.06 | 25.20 | 26.86 | 5.98 | 5.99 | 5.97 | 0.82 | 0.87 | 0.82 | 19.67 | 18.42 | 19.36 | 0.08 | 0.09 | 0.08 | 67.34 | 78.79 | 73.88 |
|  | 10-20 cm | 1.18 | 29.28 | 30.64 | 5.92 | 5.93 | 5.90 | 0.73 | 0.75 | 0.73 | 18.09 | 19.09 | 19.06 | 0.08 | 0.08 | 0.08 | 64.95 | 65.41 | 65.34 |

Note: BD, bulk density; NWC, natural water content; FMC, field moisture capacity; TP, total phosphor; TSOC, total soil organic carbon; TN, total nitrogen; ASi, available silicon.

**Supplementary table S2**: pH, TP, TN, TSOC and Asi content of sample rhizosphere soils

| *Fagopyrum*  plants | pH | | | TP  (g·kg^-1^) | | | TSOC  (g·kg^-1^) | | | TN  (g·kg^-1^) | | | ASi  (mg·kg^-1^) | | |
| --- | --- | --- | --- | --- | --- | --- | --- | --- | --- | --- | --- | --- | --- | --- | --- |
|  | 1 | 2 | 3 | 1 | 2 | 3 | 1 | 2 | 3 | 1 | 2 | 3 | 1 | 2 | 3 |
| Hongtian 5# | 5.78 | 5.77 | 5.79 | 1.05 | 0.97 | 1.02 | 22.29 | 22.25 | 21.62 | 0.12 | 0.13 | 0.11 | 82.49 | 81.30 | 84.89 |
| Hongtian 2# | 5.57 | 5.53 | 5.51 | 0.95 | 0.92 | 0.91 | 20.07 | 21.28 | 20.64 | 0.10 | 0.11 | 0.11 | 63.61 | 60.13 | 61.63 |
| Heimi-15 | 5.87 | 5.87 | 5.89 | 0.69 | 0.71 | 0.72 | 21.93 | 21.66 | 22.02 | 0.10 | 0.11 | 0.10 | 210.71 | 210.58 | 208.19 |
| Yunku 1# | 6.04 | 6.05 | 6.01 | 1.12 | 1.12 | 1.16 | 15.52 | 15.80 | 15.51 | 0.10 | 0.10 | 0.10 | 90.43 | 87.82 | 89.62 |
| Kuqiao YZX | 5.99 | 6.00 | 6.01 | 1.01 | 1.07 | 1.04 | 18.40 | 18.43 | 17.73 | 0.10 | 0.10 | 0.09 | 115.32 | 116.35 | 119.34 |
| GJK 2012-2294# | 5.73 | 5.76 | 5.78 | 1.04 | 0.98 | 1.06 | 21.96 | 22.24 | 22.60 | 0.11 | 0.12 | 0.12 | 117.41 | 119.62 | 116.41 |
| GJK 2012-2298# | 5.78 | 5.77 | 5.78 | 0.84 | 0.83 | 0.80 | 22.32 | 21.63 | 22.27 | 0.11 | 0.12 | 0.11 | 93.12 | 91.85 | 94.85 |
| GJK 2012-1206# | 6.01 | 6.03 | 6.01 | 0.72 | 0.74 | 0.76 | 21.62 | 22.60 | 21.32 | 0.11 | 0.12 | 0.11 | 102.64 | 102.75 | 102.48 |
| GMK 2010-2002# | 5.61 | 5.61 | 5.59 | 0.96 | 1.00 | 1.00 | 14.85 | 15.85 | 14.88 | 0.08 | 0.08 | 0.08 | 88.33 | 89.13 | 88.23 |
| GMK 2012-2163# | 5.85 | 5.84 | 5.84 | 0.91 | 0.95 | 0.92 | 15.85 | 15.18 | 16.14 | 0.08 | 0.09 | 0.09 | 124.42 | 112.10 | 121.67 |
| GMK 2012-19# | 5.75 | 5.76 | 5.73 | 0.89 | 0.84 | 0.86 | 16.48 | 16.17 | 16.12 | 0.10 | 0.11 | 0.11 | 85.42 | 86.84 | 83.69 |
| HXJQ | 5.68 | 5.65 | 5.69 | 0.86 | 0.84 | 0.86 | 22.91 | 22.59 | 21.90 | 0.11 | 0.10 | 0.06 | 62.28 | 62.09 | 61.52 |
| JQ 1# | 5.87 | 5.87 | 5.86 | 0.74 | 0.77 | 0.76 | 20.37 | 20.34 | 21.29 | 0.11 | 0.11 | 0.12 | 89.63 | 87.97 | 88.92 |
| JQ 3# | 5.54 | 5.54 | 5.52 | 0.64 | 0.61 | 0.59 | 20.96 | 21.34 | 20.32 | 0.10 | 0.11 | 0.10 | 65.81 | 66.24 | 65.42 |
| Low JQ | 5.76 | 5.79 | 5.77 | 0.92 | 0.95 | 0.89 | 20.71 | 21.93 | 20.36 | 0.11 | 0.11 | 0.11 | 86.24 | 83.54 | 83.29 |

**Supplementary table S2** (Continual)

| *Fagopyrum*  plants | pH | | | TP  (g·kg^-1^) | | | TSOC  (g·kg^-1^) | | | TN  (g·kg^-1^) | | | ASi  (mg·kg^-1^) | | |
| --- | --- | --- | --- | --- | --- | --- | --- | --- | --- | --- | --- | --- | --- | --- | --- |
|  | 1 | 2 | 3 | 1 | 2 | 3 | 1 | 2 | 3 | 1 | 2 | 3 | 1 | 2 | 3 |
| MYQ 1# | 5.93 | 5.92 | 5.92 | 0.74 | 0.67 | 0.71 | 21.67 | 21.66 | 21.64 | 0.10 | 0.10 | 0.10 | 67.85 | 68.63 | 68.37 |

Note: TP, total phosphor; TSOC, total soil organic carbon; TN, total nitrogen; ASi, available silicon.

**Supplementary table S3**: Total Si content of *Fagopyrum* plants studied

| *Fagopyrum*  plants | Collected time | Total Si content  (g·kg^-1^) | | | | | | | | |
| --- | --- | --- | --- | --- | --- | --- | --- | --- | --- | --- |
|  |  | Leaf | | | Stem | | | Root | | |
|  |  | 1 | 2 | 3 | 1 | 2 | 3 | 1 | 2 | 3 |
| Hongtian 5# | May | 7.199 | 7.029 | 7.207 | 5.521 | 5.73 | 5.872 | 26.194 | 29.234 | 25.921 |
| Hongtian 2# | May | 10.057 | 10.105 | 10.805 | 8.452 | 8.811 | 9.058 | 19.967 | 21.091 | 18.023 |
| Heimi-15 | May | 5.206 | 5.778 | 5.062 | 4.631 | 4.383 | 4.759 | 5.987 | 6.084 | 6.732 |
| Yunku 1# | May | 5.794 | 5.757 | 6.173 | 3.923 | 3.928 | 4.113 | 15.327 | 13.091 | 15.236 |
| Kuqiao YZX | May | 3.522 | 4.073 | 3.703 | 5.69 | 5.722 | 5.312 | 9.129 | 8.167 | 10.231 |
| GJK 2012-2294# | May | 3.497 | 3.778 | 3.129 | 3.484 | 3.063 | 3.659 | 13.331 | 14.221 | 16.268 |
| GJK 2012-2298# | May | 6.438 | 6.981 | 6.579 | 4.455 | 4.213 | 4.469 | 10.253 | 11.21 | 12.298 |
| GJK 2012-1206# | May | 3.426 | 3.971 | 3.906 | 3.887 | 3.209 | 4.023 | 15.167 | 13.197 | 12.698 |
| GMK 2010-2002# | May | 8.291 | 8.304 | 8.925 | 7.236 | 7.064 | 7.311 | 13.189 | 10.969 | 10.069 |
| GMK 2012-2163# | May | 3.998 | 3.464 | 4.003 | 4.11 | 4.407 | 4.137 | 23.334 | 24.415 | 23.098 |
| GMK 2012-19# | May | 5.718 | 5.436 | 5.739 | 5.658 | 5.096 | 5.631 | 25.931 | 28.055 | 26.061 |
| HXJQ | May | 4.507 | 4.975 | 4.873 | 3.103 | 3.701 | 3.741 | 8.049 | 8.98 | 8.001 |
| JQ 1# | May | 3.665 | 4.152 | 4.015 | 3.594 | 3.478 | 3.459 | 13.133 | 13.571 | 15.095 |
| JQ 3# | May | 3.109 | 4.094 | 4.083 | 3.369 | 3.766 | 3.882 | 5.648 | 5.358 | 5.255 |
| Low JQ | May | 4.469 | 4.211 | 4.369 | 2.922 | 2.862 | 3.106 | 14.942 | 12.463 | 12.036 |
| MYQ 1# | May | 5.606 | 5.057 | 5.592 | 4.904 | 4.826 | 5.326 | 9.088 | 9.016 | 9.635 |
| Heimi-15 | July | 9.193 | 8.761 | 8.803 | 6.017 | 5.262 | 4.153 | 23.226 | 21.494 | 25.625 |
| Kuqiao YZX | July | -- | -- | -- | 6.238 | 6.367 | 6.067 | 11.954 | 11.701 | 13.089 |
| Yunku 1# | July | -- | -- | -- | 8.017 | 8.186 | 9.059 | 18.632 | 20.081 | 16.326 |
| Tianqiao BH | July | 9.454 | 9.304 | 10.312 | 4.016 | 3.797 | 4.215 | 14.072 | 14.436 | 15.302 |
| GJK 2012-2294# | July | 6.112 | 5.323 | 5.671 | 4.834 | 4.405 | 4.056 | 12.654 | 12.491 | 14.099 |
| GJK 2012-1206# | July | 4.919 | 4.541 | 5.032 | 6.923 | 6.712 | 6.229 | 17.906 | 17.019 | 16.729 |
| Kuqiao YZX | July | 10.752 | 10.314 | 10.034 | 5.748 | 6.755 | 5.698 | 24.152 | 25.262 | 25.735 |
| GMK 2012-19# | July | -- | -- | -- | 5.864 | 5.023 | 5.402 | 30.105 | 28.261 | 31.019 |
| HXJQ | July | 5.984 | 5.972 | 5.131 | 4.399 | 4.211 | 4.769 | 14.256 | 14.734 | 16.082 |

**Supplementary table S3** (Continual)

| *Fagopyrum*  plants | Collected time | Total Si content  (g·kg^-1^) | | | | | | | | |
| --- | --- | --- | --- | --- | --- | --- | --- | --- | --- | --- |
|  |  | Leaf | | | Stem | | | Root | | |
|  |  | 1 | 2 | 3 | 1 | 2 | 3 | 1 | 2 | 3 |
| JQ 1# | July | 5.941 | 5.533 | 6.011 | 4.182 | 4.497 | 4.435 | 13.627 | 13.217 | 15.124 |
| JQ 3# | July | 4.736 | 4.964 | 4.698 | 4.931 | 4.445 | 3.823 | 9.455 | 9.645 | 9.702 |
| Low JQ | July | 6.808 | 6.841 | 6.413 | 5.888 | 6.817 | 5.463 | 22.897 | 23.986 | 25.038 |
| MYQ 1# | July | 8.939 | 8.904 | 8.864 | 6.408 | 5.055 | 6.256 | 10.098 | 10.078 | 9.852 |

**Supplementary table S4**: Phytolith content of *Fagopyrum* plants studied

| *Fagopyrum*  plants | Collected  Time | Phytolith content  (g·kg^-1^) | | | | | | | | |
| --- | --- | --- | --- | --- | --- | --- | --- | --- | --- | --- |
|  |  | Leaf | | | Stem | | | Root | | |
|  |  | 1 | 2 | 3 | 1 | 2 | 3 | 1 | 2 | 3 |
| Hongtian 5# | May | 4.3495 | 5.2825 | 4.5824 | 0.5067 | 0.6298 | 0.4166 | 44.540 | 47.802 | 43.724 |
| Hongtian 2# | May | 9.5281 | 9.8753 | 10.927 | 1.0399 | 1.1824 | 1.4566 | 17.748 | 16.754 | 18.483 |
| Heimi-15 | May | 1.6613 | 1.0119 | 1.5616 | 0.3566 | 0.4266 | 0.5933 | 2.6663 | 4.6980 | 3.4326 |
| Yunku 1# | May | 4.9491 | 4.0867 | 4.4488 | 0.1625 | 0.2800 | 0.3100 | 12.036 | 11.079 | 12.435 |
| Kuqiao YZX | May | 1.7495 | 1.8115 | 1.5492 | 0.4625 | 0.3125 | 0.2950 | 9.9485 | 9.2477 | 10.945 |
| GJK 2012-2294# | May | 1.7994 | 1.8238 | 1.3994 | 0.2560 | 0.3340 | 0.2260 | 7.6705 | 6.3225 | 8.1224 |
| GJK 2012-2298# | May | 6.1206 | 5.7084 | 6.5606 | 0.5499 | 0.5648 | 0.7100 | 7.6485 | 5.9994 | 6.7980 |
| GJK 2012-1206# | May | 1.4867 | 1.7237 | 1.9489 | 0.2825 | 0.3250 | 0.2850 | 13.198 | 14.835 | 12.042 |
| GMK 2010-2002# | May | 9.8472 | 9.2460 | 8.8986 | 1.3741 | 1.5241 | 1.6736 | 13.743 | 15.443 | 16.045 |
| GMK 2012-2163# | May | 1.6116 | 1.2245 | 1.6993 | 0.5750 | 0.6150 | 0.5299 | 23.869 | 20.666 | 21.274 |
| GMK 2012-19# | May | 3.8361 | 3.2228 | 3.9227 | 0.6049 | 0.5950 | 0.8049 | 27.781 | 27.997 | 31.197 |
| HXJQ | May | 1.7282 | 1.4857 | 1.3495 | 0.3425 | 0.3150 | 0.3849 | 4.3182 | 3.3389 | 4.3986 |
| JQ 1# | May | 1.4776 | 1.2274 | 1.1276 | 0.2775 | 0.2850 | 0.3574 | 6.5914 | 5.0220 | 4.9574 |
| JQ 3# | May | 1.0561 | 1.3430 | 1.3934 | 0.3800 | 0.3999 | 0.5266 | 2.2000 | 3.1600 | 3.2399 |
| Low JQ | May | 1.2865 | 1.6995 | 1.4531 | 0.2920 | 0.2820 | 0.4020 | 6.0309 | 7.5977 | 5.5330 |
| MYQ 1# | May | 1.9164 | 1.8243 | 1.6579 | 0.6555 | 0.6277 | 0.7721 | 4.9968 | 5.5326 | 5.7663 |
| Heimi-15 | July | 5.7213 | 5.4641 | 6.0884 | 0.4160 | 0.5000 | 0.3840 | 12.584 | 10.624 | 12.297 |
| Kuqiao YZX | July | -- | -- | -- | 0.8249 | 0.6698 | 0.6748 | 6.2368 | 3.3384 | 5.2962 |
| Yunku 1# | July | -- | -- | -- | 1.5192 | 1.4398 | 1.5492 | 11.897 | 11.295 | 10.866 |
| Tianqiao BH | July | 6.7376 | 7.1874 | 6.2485 | 0.4700 | 0.3733 | 0.4533 | 9.9498 | 8.1228 | 8.6267 |
| GJK 2012-2294# | July | 0.8720 | 0.5886 | 1.0994 | 0.5875 | 0.5832 | 0.5956 | 17.763 | 18.448 | 21.166 |
| GJK 2012-1206# | July | 0.6498 | 0.8059 | 0.9498 | 1.4636 | 1.6788 | 1.5592 | 20.389 | 19.726 | 22.118 |
| Kuqiao YZX | July | 13.670 | 12.855 | 14.569 | 1.2891 | 1.4297 | 1.3300 | 35.994 | 37.521 | 38.726 |
| GMK 2012-19# | July | -- | -- | -- | 1.2791 | 1.4294 | 1.3496 | 68.911 | 71.514 | 65.701 |

**Supplementary table S4** (Continual)

| *Fagopyrum*  plants | Collected  Time | Phytolith content  (g·kg^-1^) | | | | | | | | |
| --- | --- | --- | --- | --- | --- | --- | --- | --- | --- | --- |
|  |  | Leaf | | | Stem | | | Root | | |
|  |  | 1 | 2 | 3 | 1 | 2 | 3 | 1 | 2 | 3 |
| HXJQ | July | 1.6645 | 2.0662 | 1.9995 | 0.5733 | 0.4166 | 0.3933 | 19.871 | 19.028 | 18.499 |
| JQ 1# | July | 1.8498 | 2.1098 | 1.5598 | 0.4720 | 0.4600 | 0.4959 | 16.630 | 17.088 | 18.423 |
| JQ 3# | July | 3.7477 | 4.2987 | 4.4834 | 0.8200 | 0.8465 | 0.8064 | 11.355 | 14.450 | 11.864 |
| Low JQ | July | 6.0813 | 5.8310 | 6.1467 | 0.9998 | 1.0831 | 0.9664 | 28.640 | 32.538 | 31.154 |
| MYQ 1# | July | 13.176 | 13.067 | 14.440 | 1.5245 | 1.3497 | 1.7249 | 13.340 | 15.747 | 14.465 |

**Supplementary table S5**: PhytOC content of *Fagopyrum* plants studied

| *Fagopyrum*  plants | Collected  time | PhytOC content  (g·kg^-1^) | | | | | | | | |
| --- | --- | --- | --- | --- | --- | --- | --- | --- | --- | --- |
|  |  | Leaf | | | Stem | | | Root | | |
|  |  | 1 | 2 | 3 | 1 | 2 | 3 | 1 | 2 | 3 |
| Hongtian 5# | May | 0.2828 | 0.3284 | 0.3425 | 0.0363 | 0.0472 | 0.0315 | 0.5132 | 0.5531 | 0.4819 |
| Hongtian 2# | May | 0.7953 | 0.7798 | 0.8752 | 0.1000 | 0.1180 | 0.1175 | 0.5327 | 0.5027 | 0.5552 |
| Heimi-15 | May | 0.0878 | 0.0520 | 0.0818 | 0.0299 | 0.0342 | 0.0424 | 0.0357 | 0.0711 | 0.0523 |
| Yunku 1# | May | 0.4176 | 0.3642 | 0.3981 | 0.0075 | 0.0128 | 0.0142 | 0.1246 | 0.1314 | 0.1480 |
| Kuqiao YZX | May | 0.0731 | 0.0859 | 0.0567 | 0.0339 | 0.0252 | 0.0267 | 0.1025 | 0.1042 | 0.1220 |
| GJK 2012-2294# | May | 0.0579 | 0.0586 | 0.0398 | 0.0256 | 0.0364 | 0.0226 | 0.1297 | 0.1067 | 0.1367 |
| GJK 2012-2298# | May | 0.5359 | 0.4831 | 0.5988 | 0.0613 | 0.0627 | 0.0799 | 0.1126 | 0.0879 | 0.0979 |
| GJK 2012-1206# | May | 0.1333 | 0.1532 | 0.1729 | 0.0323 | 0.0390 | 0.0312 | 0.1706 | 0.1851 | 0.1506 |
| GMK 2010-2002# | May | 0.5198 | 0.5416 | 0.4814 | 0.1120 | 0.1175 | 0.1348 | 0.0928 | 0.0936 | 0.1111 |
| GMK 2012-2163# | May | 0.1142 | 0.0938 | 0.1459 | 0.0324 | 0.0358 | 0.0397 | 0.6271 | 0.5612 | 0.5320 |
| GMK 2012-19# | May | 0.2459 | 0.1895 | 0.2322 | 0.0180 | 0.0179 | 0.0309 | 0.2985 | 0.3070 | 0.3274 |
| HXJQ | May | 0.0841 | 0.0738 | 0.0550 | 0.0477 | 0.0414 | 0.0484 | 0.1193 | 0.0922 | 0.1208 |
| JQ 1# | May | 0.1188 | 0.0978 | 0.0826 | 0.0517 | 0.0521 | 0.0631 | 0.1077 | 0.0816 | 0.0800 |
| JQ 3# | May | 0.0425 | 0.0534 | 0.0614 | 0.0272 | 0.0270 | 0.0347 | 0.0294 | 0.0440 | 0.0479 |
| Low JQ | May | 0.0496 | 0.0678 | 0.0521 | 0.0273 | 0.0239 | 0.0360 | 0.1040 | 0.1332 | 0.0967 |
| MYQ 1# | May | 0.0976 | 0.0958 | 0.0718 | 0.0504 | 0.0488 | 0.0567 | 0.0696 | 0.0754 | 0.0787 |
| Heimi-15 | July | 0.5667 | 0.5496 | 0.6059 | 0.0646 | 0.0728 | 0.0531 | 0.1240 | 0.0955 | 0.0714 |
| Kuqiao YZX | July | -- | -- | -- | 0.0623 | 0.0574 | 0.0669 | 0.2499 | 0.1345 | 0.1996 |
| Yunku 1# | July | -- | -- | -- | 0.1005 | 0.0853 | 0.0983 | 0.4436 | 0.4155 | 0.3368 |
| Tianqiao BH | July | 0.7106 | 0.7568 | 0.6817 | 0.0100 | 0.0059 | 0.0122 | 0.0716 | 0.0734 | 0.0443 |
| GJK 2012-2294# | July | 0.0626 | 0.0468 | 0.0760 | 0.0719 | 0.0699 | 0.0794 | 0.2097 | 0.2089 | 0.2378 |
| GJK 2012-1206# | July | 0.0797 | 0.0917 | 0.1027 | 0.1472 | 0.1675 | 0.1416 | 0.3408 | 0.3011 | 0.3736 |
| Kuqiao YZX | July | 0.5534 | 0.5117 | 0.5583 | 0.0968 | 0.1144 | 0.1064 | 2.0375 | 2.0252 | 2.1881 |
| GMK 2012-19# | July | -- | -- | -- | 0.0767 | 0.0916 | 0.0911 | 1.6313 | 1.6950 | 1.5606 |
| HXJQ | July | 0.2949 | 0.3963 | 0.3601 | 0.1010 | 0.0785 | 0.0780 | 0.2078 | 0.1974 | 0.1420 |

**Supplementary table S5** (Continual)

| *Fagopyrum*  plants | Collected  time | PhytOC content  (g·kg^-1^) | | | | | | | | |
| --- | --- | --- | --- | --- | --- | --- | --- | --- | --- | --- |
|  |  | Leaf | | | Stem | | | Root | | |
|  |  | 1 | 2 | 3 | 1 | 2 | 3 | 1 | 2 | 3 |
| JQ 1# | July | 0.1718 | 0.1860 | 0.1327 | 0.0771 | 0.0717 | 0.0866 | 0.1888 | 0.1750 | 0.1430 |
| JQ 3# | July | 0.3319 | 0.4175 | 0.3441 | 0.1276 | 0.1348 | 0.1324 | 0.1812 | 0.2367 | 0.1897 |
| Low JQ | July | 0.9904 | 0.9064 | 0.9164 | 0.2325 | 0.2455 | 0.2182 | 0.1816 | 0.2093 | 0.1583 |
| MYQ 1# | July | 1.3439 | 1.3968 | 1.5195 | 0.2307 | 0.2135 | 0.2637 | 0.2467 | 0.2938 | 0.2675 |

Note: PhytOC, phytolith occluded organic carbon.
